# Unveiling the Antiaging Power of Rhodoxanthin From *Potamogeton crispus* L. in D‐Galactose‐Induced Aging Mice

**DOI:** 10.1002/fsn3.70379

**Published:** 2025-06-08

**Authors:** Yichao Ma, Cong Fu, Hongchun Dong, Xuhui Lei, Yuxin Shi, Yunhai He, Shu Liu, Qiukuan Wang, Dandan Ren

**Affiliations:** ^1^ College of Food Science and Engineering Dalian Ocean University Dalian People's Republic of China; ^2^ National R & D Branch Center for Seaweed Processing Dalian People's Republic of China; ^3^ Key Laboratory of Aquatic Product Processing and Utilization of Liaoning Province Dalian People's Republic of China

**Keywords:** antiaging, carotenoid, D‐galactose, rhodoxanthin

## Abstract

Anti‐aging research represents a significant and challenging frontier in biomedical science. While rhodoxanthin, a naturally occurring carotenoid, has demonstrated preliminary antioxidant properties, its precise antiaging mechanisms remain poorly understood. This study systematically investigated the antiaging effects of rhodoxanthin in a D‐galactose‐induced murine aging model with particular focus on elucidating its underlying molecular mechanisms. Our findings revealed that rhodoxanthin administration significantly attenuated oxidative damage in both brain and liver tissues, as evidenced by reduced lipid peroxidation and enhanced activities of key antioxidant enzymes. At the optimal dosage (80 mg/kg rhodoxanthin), antioxidant enzyme activities were restored to 84.3% (CAT), 66.7% (SOD), and 145% (GPX) of model control levels in the liver and 61.36% (CAT), 4.2% (SOD), and 22.2% (GPX) in the brain. Mechanistic studies indicated that rhodoxanthin's antiaging effects were mediated through modulation of the nuclear factor erythroid‐related factor ‌Nrf2 and PI3K/Akt signaling pathways. Quantitative analysis demonstrated significant upregulation of Nrf2, PI3K, and Akt expression in both hepatic and cerebral tissues of aging mice, and behavioral assessments confirmed that rhodoxanthin not only served as a potent natural antioxidant but also improved memory retention and cognitive function in aged subjects. These results collectively established rhodoxanthin as food‐functional component, with dual protective effects against both oxidative damage and cognitive decline. Rhodoxanthin could be used as a natural antioxidant in the food industry.

## Graphical abstract

Rhodoxanthin prevents the decrease in antioxidant enzyme activity. Rhodoxanthin can significantly up‐regulate the expression levels of Nrf2, PI3K, and Akt. Rhodoxanthin can act as an antiaging agent.z

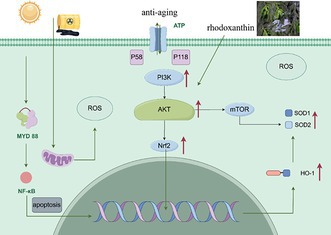

**Abbreviations**
Akt: Protein kinase B
CAT: Catalase
GPX: Glutathione peroxidase
GSH: Reduced glutathione
HO‐1: Interleukin‐10
HPLC: High‐performance liquid chromatography
LDH: Lactate dehydrogenase
MDA: Malonaldehyde
MS: Mass spectrometry
PI3K: Phosphplnositide‐3 kinase
ROS: Active oxygen
SOD: Superoxide dismutase
SOD1: Superoxide dismutase 1
SOD2: Superoxide dismutase 2
UV–Vis: Visible spectrophotometry

## 1 Introduction

The global population is currently experiencing unprecedented aging, a demographic trend accompanied by progressive deterioration of cellular and organ function that significantly elevates susceptibility to age‐related pathologies, including cardiovascular diseases and cognitive decline (Shen et al. 2017). Contemporary research identified that oxidative stress, characterized by excessive accumulation of reactive oxygen species (ROS), is the primary molecular driver of aging processes (Hu et al. 2022; Yu and Xiao 2021). During normal aerobic metabolism in aging organisms, ROS production is escalated while endogenous antioxidant defense mechanisms become progressively impaired (Zhang et al. 2020). This imbalance between oxidative stress and antioxidant capacity leads to cumulative oxidative damage to cellular components, ultimately causing irreversible tissue damage, accelerated senescence, and the development of degenerative disorders (Luo et al. 2020).

The endogenous antioxidant defense system, comprising both enzymatic and non‐enzymatic components, plays a crucial role in protecting organisms against free radical‐induced damage through the elimination of excess ROS (Hernandez et al. 2021). Increasing evidence suggests a strong correlation between the aging process and diminished antioxidant capacity. Therapeutic strategies involving antioxidant supplementation have emerged as a promising approach to counteract age‐related oxidative damage by neutralizing excessive free radicals. Recent studies have demonstrated that rhodoxanthin, a bioactive compound isolated from *Potamogeton crispus L*., exhibits significant cytoprotective effects against oxidative stress. Notably, Fu et al. (2023) reported that rhodoxanthin treatment enhanced the activities of key antioxidant enzymes (SOD, CAT, and GPX) while reducing glutathione (GSH) depletion in H_2_O_2_‐induced cellular damage models. Furthermore, it effectively decreased intracellular lactate dehydrogenase (LDH) activity, malondialdehyde (MDA) content, and ROS levels. While numerous synthetic compounds have demonstrated antiaging properties, their clinical application is often limited by adverse effects associated with prolonged use, including immunosuppression and hypertension (Kong et al. 2018). Consequently, natural antioxidants derived from plant sources have garnered significant attention in gerontological research. Current evidence supports that dietary supplementation with plant‐based antioxidants can effectively ameliorate oxidative stress, thereby attenuating the aging process through multiple molecular mechanisms.

**FIGURE 1:**
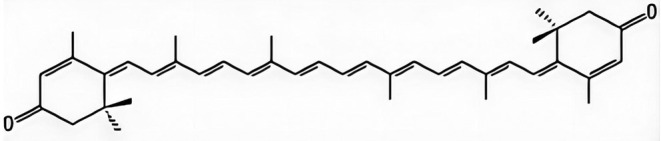
The structure of rhodoxanthin.

Carotenoids, a class of naturally occurring pigments widely distributed in biological systems, play vital roles in numerous physiological processes (Eroglu et al. 2023). These compounds exhibit potent antioxidant properties, effectively scavenging free radicals and protecting cellular components from oxidative damage. Additionally, carotenoids demonstrated immunomodulatory effects through their ability to regulate various immune cell functions, thereby enhancing host defense mechanisms (Kurniawan et al. 2023). *Potamogeton crispus L*., a perennial submerged macrophyte with global distribution, represents an important wild resource commonly found in lentic ecosystems such as lakes and reservoirs (Ren and Zhang 2008). Recent phytochemical analyses have identified rhodoxanthin as the principal red carotenoid in this aquatic plant (Fu et al. 2023; structure shown in Figure 1). Accumulating evidence indicates that rhodoxanthin possesses diverse biological activities, particularly notable antioxidant and immunostimulatory properties. Experimental studies have demonstrated that rhodoxanthin effectively inhibited malondialdehyde production and scavenged hydroxyl radicals, thereby preventing hydroxyl radical‐induced lipid peroxidation in murine hepatic mitochondria (Ren et al. 2006). The pathological accumulation of intracellular ROS can overwhelm endogenous antioxidant defense systems, resulting in oxidative damage to critical biomolecules including proteins and carbohydrates. Such molecular damage ultimately led to cellular dysfunction and apoptosis (Höhn et al. 2017). When ROS generation exceeded the scavenging capacity of physiological antioxidant systems, tissues experienced significant peroxidative damage. Notably, rhodoxanthin exhibited promising anticancer properties, as evidenced by its ability to suppress proliferation and induce apoptosis in human cervical cancer cells. Characteristic morphological changes including nuclear fragmentation, chromatin condensation, and cell shrinkage have been observed in rhodoxanthin‐treated cancer cells (Ren et al. 2006). These findings collectively suggested that rhodoxanthin may serve as a potential therapeutic agent through its pro‐apoptotic effects on malignant cells.

D‐galactose has been shown to accelerate oxidative damage by promoting the generation of oxygen‐derived free radicals, which subsequently degrade cellular macromolecules and compromise tissue integrity (Hu et al. 2022). The D‐galactose‐induced aging mouse model closely recapitulates both morphological and molecular characteristics of natural aging, making it a well‐established system for investigating aging processes. In this study, we isolated the bioactive red carotenoid rhodoxanthin from *Potamogeton crispus* L. and employed an intraperitoneal D‐galactose injection protocol to establish an oxidative stress‐mediated aging model in mice. Through comprehensive assessment of oxidative stress markers, hepatic and cerebral damage indicators, and key components of oxidative signaling pathways, we systematically evaluate the antiaging efficacy of rhodoxanthin and elucidate its underlying mechanisms of action.

## 2 Materials and Methods

### 2.1 Chemicals and Materials

KM mice were procured from Liaoning Changsheng Biotechnology Co. Ltd. Commercial assay kits for serum biochemical analysis, including SOD, CAT, and MDA detection, were obtained from Nanjing Jiancheng Bioengineering Institute.

### 2.2 Structural Analysis

Silica gel (200–300 mesh) was employed for column chromatography after activation at 120°C for 2 h. The crude extracts were fractionated using gradient elution with solvent systems of varying polarity. Distinct pigment bands were collected, concentrated under reduced pressure at 40°C, and stored in acetone at −18°C. For thin‐layer chromatography (TLC), we employed a solvent system comprising n‐hexane: ethyl acetate:acetone:methanol (27:4:2:2, v/v/v/v) to separate rhodoxanthin isomers (designated r1, r2, and r3). HPLC analysis was performed using a ZORBAX SB‐C18 column (4.6 × 150 mm, 5 μm) with acetonitrile: methanol (70:30) mobile phase at 0.5 mL/min flow rate. The detection wavelength was set at 480 nm with a 15 min run time. Structural confirmation was achieved using an Agilent Q‐TOF 6540 mass spectrometer.

### 2.3 Animals and Treatments

**FIGURE 2:**
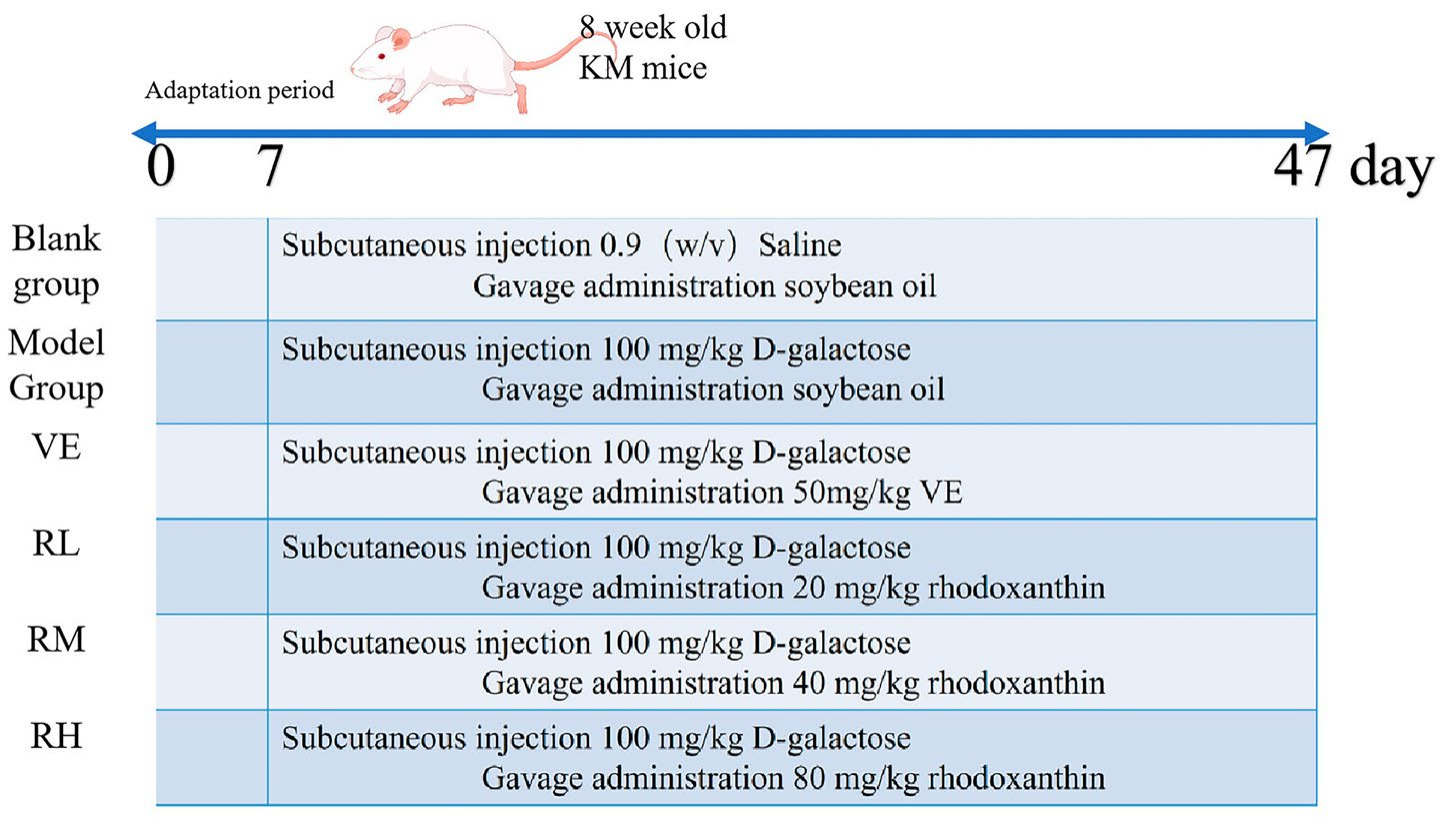
Illustrative diagrams of animal experimental procedures. RH, high doses of rhodoxanthin; RL, low doses of rhodoxanthin; RM, moderate doses of rhodoxanthin.

SPF‐grade male KM mice (18–22 g) were procured from Liaoning Changsheng Biotechnology Co. Ltd. The animals were housed under controlled environmental conditions, maintaining a constant temperature of 25°C ± 1°C with adequate ventilation and a 12‐h light/dark cycle. Prior to experimental procedures, the mice were provided with ad libitum access to food and water. The experimental protocol was conducted as illustrated in Figure 2.

### 2.4 Assessment of General Appearance, Body Weight, and Relative Organ Weight

Throughout the experimental period, the general appearance of the animals was monitored weekly. Daily records of food and water intake were maintained, and body weight was measured every 3 days. The administered dosage for each animal was calculated based on its body weight and adjusted periodically to account for weight fluctuations.

After 40 days of intragastric administration, the mice were euthanized following a 12‐h fasting period. Blood samples were collected via enucleation of the eyeballs. Subsequently, a necropsy was performed to excise key organs, including the liver, kidneys, brain, and heart. The harvested organs were rinsed with normal saline, weighed, and used to calculate the organ‐to‐body weight ratio (organ index).

$$\text{Organ index}\left(\%\right)=\frac{\text{Organ weight}\left(g\right)}{\text{Mice weight}\left(g\right)}\times 100\%$$

### 2.5 Biochemical Analysis

A precisely weighed portion of liver tissue was excised from the fixed region of the left hepatic lobe. The tissue was rinsed with ice‐cold normal saline to remove residual blood and subsequently blotted dry using filter paper. The liver samples were then homogenized under controlled conditions. A section of the murine liver was dissected and fixed in formalin for further histological processing, while the remaining tissue was rapidly frozen in liquid nitrogen and stored at −80°C for subsequent analysis.

For brain tissue processing, the right hippocampus was carefully isolated and homogenized in a 10‐fold volume of ice‐cold 0.9% normal saline. The resulting homogenate was centrifuged at 4000 × g for 10 min at 4°C to obtain the supernatant, which was aliquoted and stored at –80°C for biochemical assays.

Key oxidative stress markers, including MDA levels, as well as the enzymatic activities of SOD, CAT, and GPX, were quantitatively analyzed in the serum, liver, and brain tissues of the experimental mice.

### 2.6 Histopathological Analysis

Liver and brain tissues were harvested from mice, and residual blood was removed by rinsing with normal saline. The tissues were then fixed by immersion in formalin for at least 12 h. Subsequently, the tissues were cleared by immersion in xylene to enhance transparency. The dehydrated and cleared tissues were embedded in paraffin wax. After complete solidification, the paraffin‐embedded tissues were sectioned into small blocks and sliced into thin sections using a microtome. The sections were floated in water to ensure proper spreading and then mounted onto protein‐glycerin‐coated slides, ensuring no bubbles or impurities were trapped between the tissue sections and the slides. The slides were dried in an oven at 38°C before staining. For staining, the tissue sections were dewaxed in xylene, rehydrated through a graded ethanol series, and finally stained for microscopic examination.

### 2.7 Quantitative Real‐Time RT‐PCR Analysis

Total RNA used an RNA extraction kit (Hunan Precision Biotechnology Co. Ltd.). Subsequently, the extracted RNA was reverse‐transcribed into complementary DNA (cDNA) with a reverse transcription kit (China Precision Biotechnology (Hunan) Co. Ltd.). The purity (OD260/OD280 ratio at 1.8–2.0) and integrity (appearance of the 5S band, clear 18S and 28S bands) of the RNA samples were measured by using a BioAnalyzer and 1% agarose gel electrophoresis. Gene‐specific primers for Nrf2, HO‐1, SOD1, and SOD2 were designed (Table 1), and the expression levels of the target genes were quantified using the SYBR green‐based fluorescent method. *β‐*actin was used as the internal reference gene. The quantification cycle (*Cq*) values of both the target and reference genes were determined by quantitative real‐time RT‐PCR. The relative expression levels of the target genes, normalized to *β‐*actin, were calculated by the 2^−ΔΔCq^ method, which is a widely used approach in quantitative PCR to calculate relative gene expression between samples.

**TABLE 1:** Primer design.

Gene	Sequence content (5’ to 3’)
Nrf‐2	F: TCCGCTGCCATCAGTGAGTC
	R: ATTGTGCCTTCAGCGTGTTC
HO‐1	F: TGCAGGTGATGCTGACAGAGG
	R: ACAGACACACCCAGAACCAAA
SOD1	F: AGCATTGCCCCATCATTGGCCGTA
	R: TACTGCGCAATCCCAATCACTC
SOD2	F: TCCCAGACCTGCCTTACGA
	R: TCGGTGGCGTTGAGATGG
PI3K	F: CTACTGTAGCCAACAGCATGAA
	R: AAGGTCCCATCAGCAGTGTCTC
Akt	F: CATCGTGTGGCAGGATGTGTA
	R: ACCTGGTGTCAGTCTCAGAGGTG
*β*‐Actin	F: CATCCGTAAAGACCTCTATGCCAAC
	R: ATGGAGCCACCGATCCACA

### 2.8 Protein Expression Analysis

The protein expression levels of PI3K, Akt, and Nrf2 in brain tissues were quantified by Western blot analysis. Mouse brain tissues were rinsed two to three times with precooled PBS buffer, minced into small fragments, and homogenized in ice‐cold lysis buffer (10× tissue volume) using an ice‐bath homogenization technique. Following 30 min of ice‐bath incubation with intermittent pipetting to ensure complete lysis, the samples were centrifuged at 12,000 × g for 5 min at 4°C to obtain the tissue supernatant containing total protein extracts.

The protein lysates were mixed with loading buffer, vortexed thoroughly, and denatured by heating at 98°C for 10 min. After brief centrifugation, the denatured proteins were loaded onto SDS‐PAGE gels. Discontinuous gels were prepared according to protein molecular weights, consisting of a 10% separating gel and a 5% stacking gel. Polymerization was initiated by TEMED addition, followed by immediate gel casting. Electrophoresis was performed at constant voltages (80 V for stacking gel and 130 V for separating gel) until the dye front reached the gel bottom.

Subsequent to electrophoresis, proteins were transferred to PVDF membranes for immunoblotting. The membranes were blocked with an appropriate buffer, followed by sequential incubation with primary and secondary antibodies with gentle agitation. Finally, protein bands were visualized using enhanced chemiluminescence detection.

### 2.9 Statistical Analysis

Results were expressed as mean ± SD of triplicate measurements unless otherwise specified. Significance analysis was performed by one‐way ANOVA, with *p* < 0.05 indicating a significant difference and *p* < 0.01 indicating an extremely significant difference. Variable correlations were assessed using Pearson's or Spearman's tests, with data normality determined by Shapiro–Wilk tests.

## 3 Results

### 3.1 Structural Analysis of Rhodoxanthin

**FIGURE 3:**
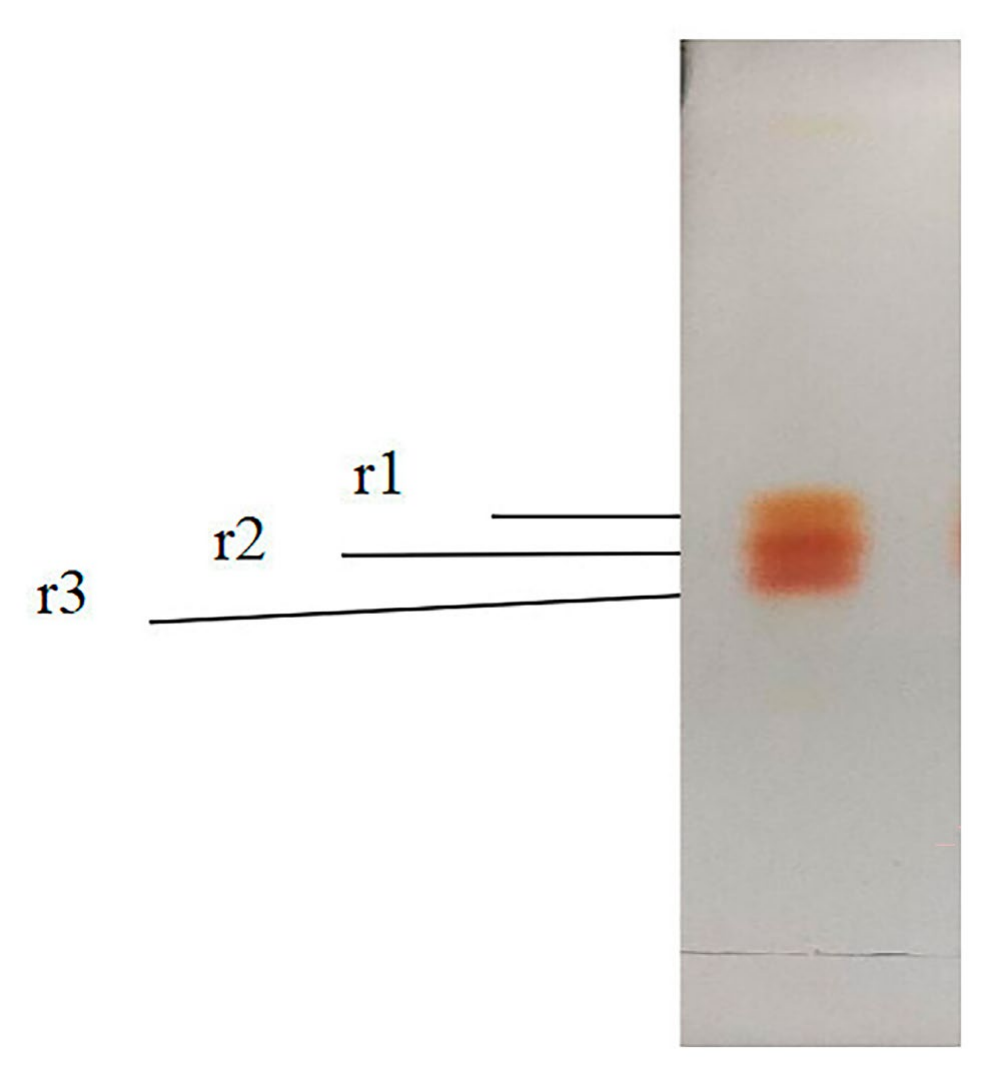
TLC of the r1, r2, and r3 from rhodoxanthin.

The red carotenoids isolated from the silica gel column were subjected to separation via silica gel G TLC, resulting in the formation of three distinct bands. As illustrated in Figure 3, these bands reflected the molecular polarity of the red carotenoids, which were consequently divided into three components based on their separation and adsorption characteristics on the silica gel G thin‐layer plate. These components were categorized according to their polarity as r1, r2, and r3.

**FIGURE 4:**
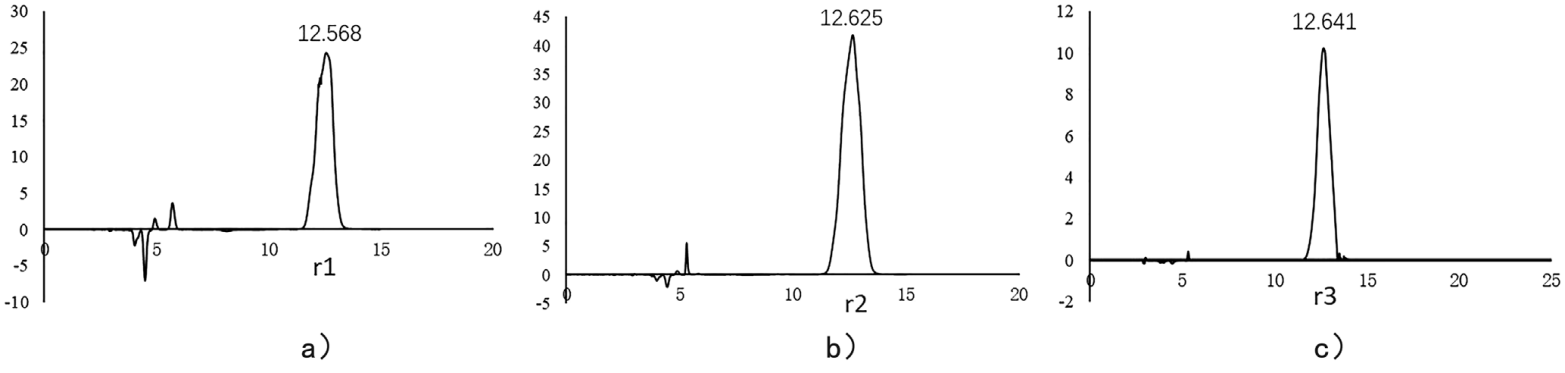
Purity test of the r1, r2, and r3 by HPLC.

The HPLC analysis of the rhodoxanthin components r1, r2, and r3 revealed distinct chromatographic peaks for each component (Figure 4). Under gradient elution conditions, the retention times for r1, r2, and r3 were recorded as 12.568, 12.625, and 12.641 min, respectively, indicating close yet distinct separation. These findings suggested that the polarity order of the components was r1>r2>r3, corroborating the results obtained from TLC.

**FIGURE 5:**
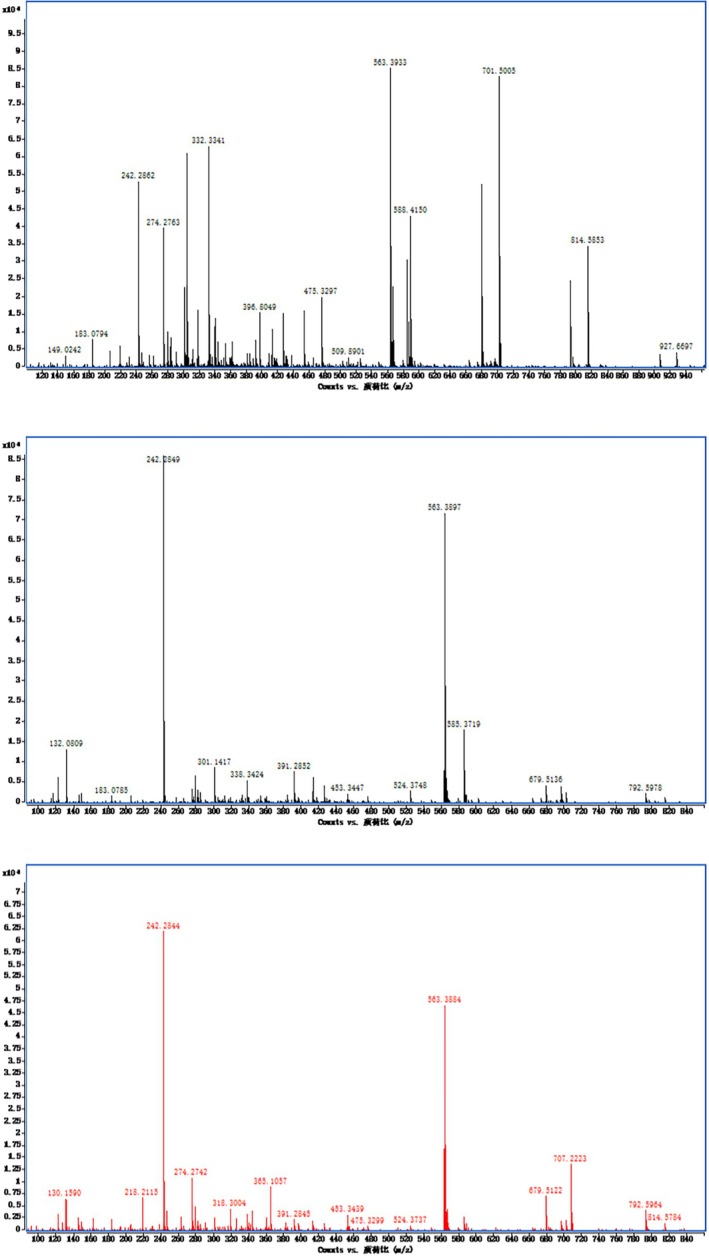
Mass spectrometry of r1, r2, and r3.

Figure 5 presented the mass spectra of the red carotenoid components r1, r2, and r3 from *Potamogeton crispus* , analyzed under electrospray ionization (ESI) in positive ionization mode. For components r1, r2, and r3, the characteristic fragment molecular peaks of carotenoids were observed (Table 2). Additionally, the mass spectrum revealed a prominent excimer ion peak at m/z 563.2933 [M + H]^+^ in r1, m/z 563.3897 [M + H]^+^ along with peaks at m/z 585.3719 [M + Na]^+^ in r2 and m/z 563.3844 [M + H]^+^ in r3. It was inferred that the relative molecular masses of r1, r2, and r3 components were all 562. These findings suggested that although the chemical structures of the compounds were similar, their fragment peaks and intensities differed. It could be deduced that r1, r2, and r3 were isomers of rhodoxanthin; the results were consistent with Schex et al. (2020). Given the extraction yield and experimental dosage requirements of rhodoxanthin, the mixture comprising rhodoxanthin r1, r2, and r3 was subsequently employed in in vivo experiments.

**TABLE 2:** Molecular ions and characteristic fragments of rhodoxanthin.

Rhodoxanthin	m/z		
	Molecular ion [M + H]^+^	Molecular ion [M + Na]^+^	Fragments (carotenoid)
r1	563.3933		149.0242, 242.2862, 332.3341
r2	563.3897	585.3719	242.2849, 338.3424, 453.3447
r3	563.3884		242.2844, 365.1057, 453.3439

### 3.2 Effects of Rhodoxanthin on Body Weight and Organ Coefficients

The changes in body weight and organ index of mice in the blank group, model group, VE group, RL group, RM group, and RH group are shown in Table 3.

**TABLE 3:** Effects of rhodoxanthin on body weight and organ coefficients of mice.

Group	Weight		Organ index (%)			
	Initial	Final	Heart	Liver	Brain	Kidney
NC	27.97 ± 0.76	36.77 ± 2.72^##^	0.70 ± 0.03^##^	3.76 ± 0.12^##^	1.13 ± 0.01^##^	1.35 ± 0.05^##^
MC	27.91 ± 0.43	34.03 ± 1.66**	0.48 ± 0.01**	3.05 ± 0.14**	0.99 ± 0.02**	1.23 ± 0.03**
VE	28.27 ± 1.14	37.29 ± 1.90^##^	0.56 ± 0.03^##^	3.50 ± 0.03^##^	1.17 ± 0.01^##^	1.34 ± 0.04^##^
RL	27.64 ± 1.72	34.04 ± 1.46	0.52 ± 0.03	3.31 ± 0.03^##^	1.10 ± 0.01	1.33 ± 0.03^##^
RM	27.48 ± 0.71	34.52 ± 1.13	0.57 ± 0.02^##^	3.48 ± 0.09^##^	1.15 ± 0.03^##^	1.35 ± 0.03##
RH	27.81 ± 0.80	37.29 ± 1.12^##^	0.64 ± 0.01^##^	3.60 ± 0.05^##^	1.16 ± 0.01^##^	1.38 ± 0.04^##^

*Note:* Compared with the blank group, **p < 0.01; compared with the model group, ##*p* < 0.01.

Abbreviations: MC, Model control group; NC, blank control group; RL, RM, and RH, low, medium, and high dose groups of rhodoxanthin; VE, positive control group.

There was no significant difference in initial body weight among all groups (*p* > 0.05). After 40 days of injection and gavage, the body weight of MC was significantly lower than that of NC (*p* < 0.01). The body weight of the RH group was significantly higher than that of MC and was close to that of NC (*p* < 0.01). The body weight of the VE group was also significantly higher than that of MC (*p* < 0.01).

The organ index directly reflects the degree of disease and nutritional status of the animal's organs. The function of tissues and organs will decline with the aging of the animal, and the atrophy of the heart, liver, brain, kidney, and other tissues and organs can well reflect the aging of the body. The organ index of the heart, liver, brain, and kidney in the MC group was significantly decreased (*p* < 0.01), and the organ index of the MC group was increased to varying degrees after different doses of rhodoxanthin, especially in the RH group. Among them, the VE group also significantly improved the organ atrophy of aging mice. This displayed that rhodoxanthin had a good protective effect on the heart, liver, brain, kidney, and other tissues and organs of aging mice.

### 3.3 Effects of Rhodoxanthin on Antioxidant Activity in Brain and Liver Tissues

**FIGURE 6:**
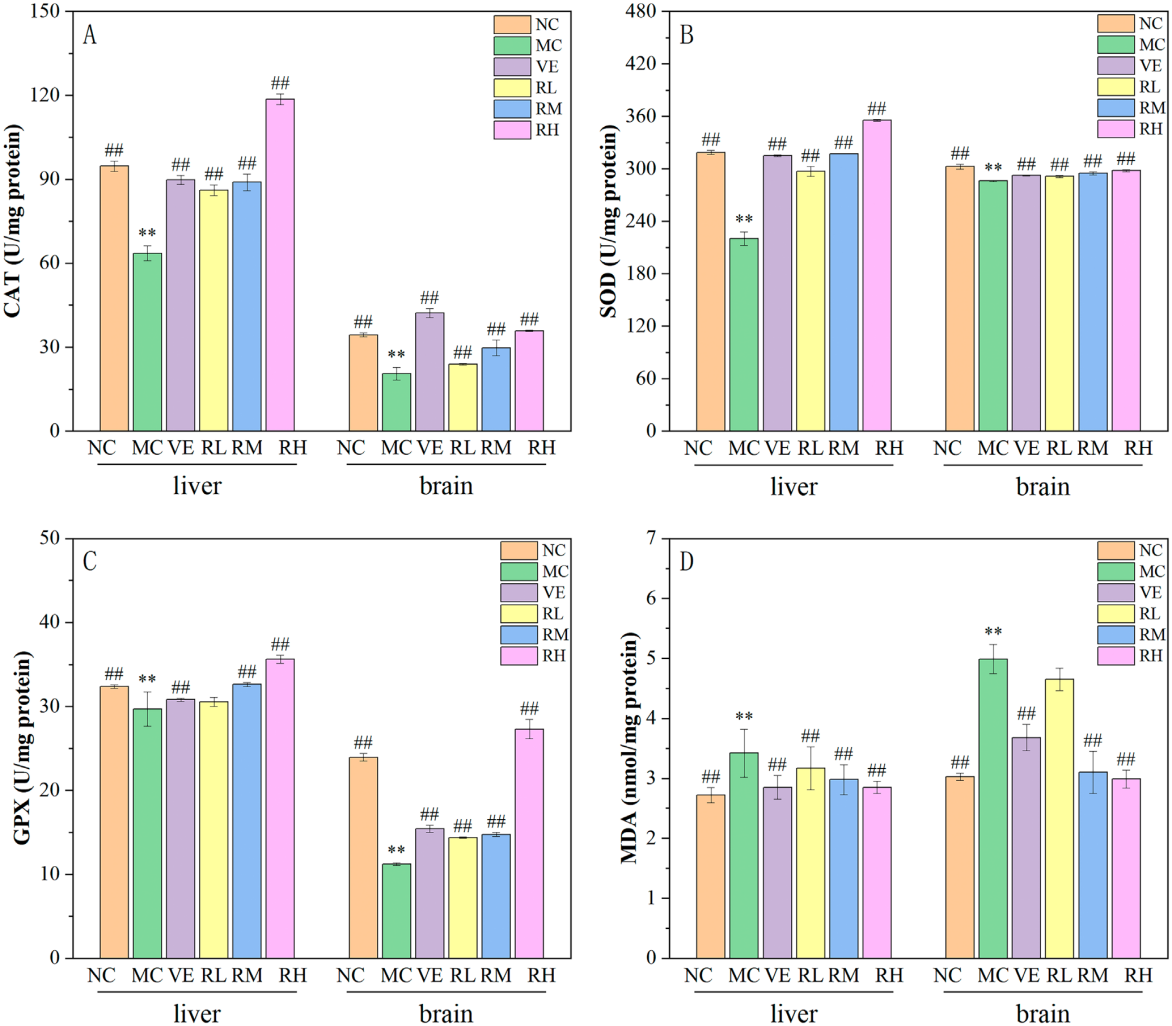
Effects of rhodoxanthin on oxidative‐related indicators (CAT, SOD, GPX, and MDA) in liver and brain MC, model control group; NC, blank control group; RL, RM, and RH, low‐, medium‐, and high‐dose groups of rhodoxanthin; VE, positive control group. Compared with the blank group (NC), **p < 0.01, compared with the model group (MC), ##*p* < 0.01.

Figure 6 demonstrates the regulatory effects of rhodoxanthin on antioxidant biomarkers (CAT, SOD, and GPX) and lipid peroxidation marker (MDA) in D‐galactose (D‐Gal)‐induced aging mice in the liver and brain. Compared with the NC group, the MC group exhibited significant reductions in CAT, SOD, and GPX activities across both hepatic and cerebral tissues (*p < 0.01*), accompanied by a marked elevation in MDA levels (*p < 0.01*), confirming the successful establishment of oxidative stress‐mediated aging. Rhodoxanthin administration dose‐dependently ameliorated these D‐Gal‐induced alterations. At the optimal dosage (80 mg/kg rhodoxanthin), antioxidant enzyme activities were restored to 84.3% (CAT), 66.7% (SOD), and 145% (GPX) of model control levels in the liver, and 61.36% (CAT), 4.2% (SOD), and 22.2% (GPX) in the brain. Notably, RH supplementation surpassed the VE positive control in enhancing CAT and SOD activities (*p < 0.05*). Concurrently, the RH group significantly attenuated oxidative damage, reducing MDA content by 40.0% in the liver and 16.6% in the brain (*p < 0.01*), outperforming the VE group's 22.2% reduction. These findings demonstrated that 80 mg/kg rhodoxanthin effectively counteracted D‐Gal‐induced oxidative aging through dual mechanisms, enzymatic antioxidant potentiation (upregulating CAT/SOD/GPX activities beyond VE's capacity), and lipid peroxidation suppression (achieving superior MDA reduction efficacy).

### 3.4 Analysis of HE Staining Results in Liver and Brain Tissue of Aging Mice

**FIGURE 7:**
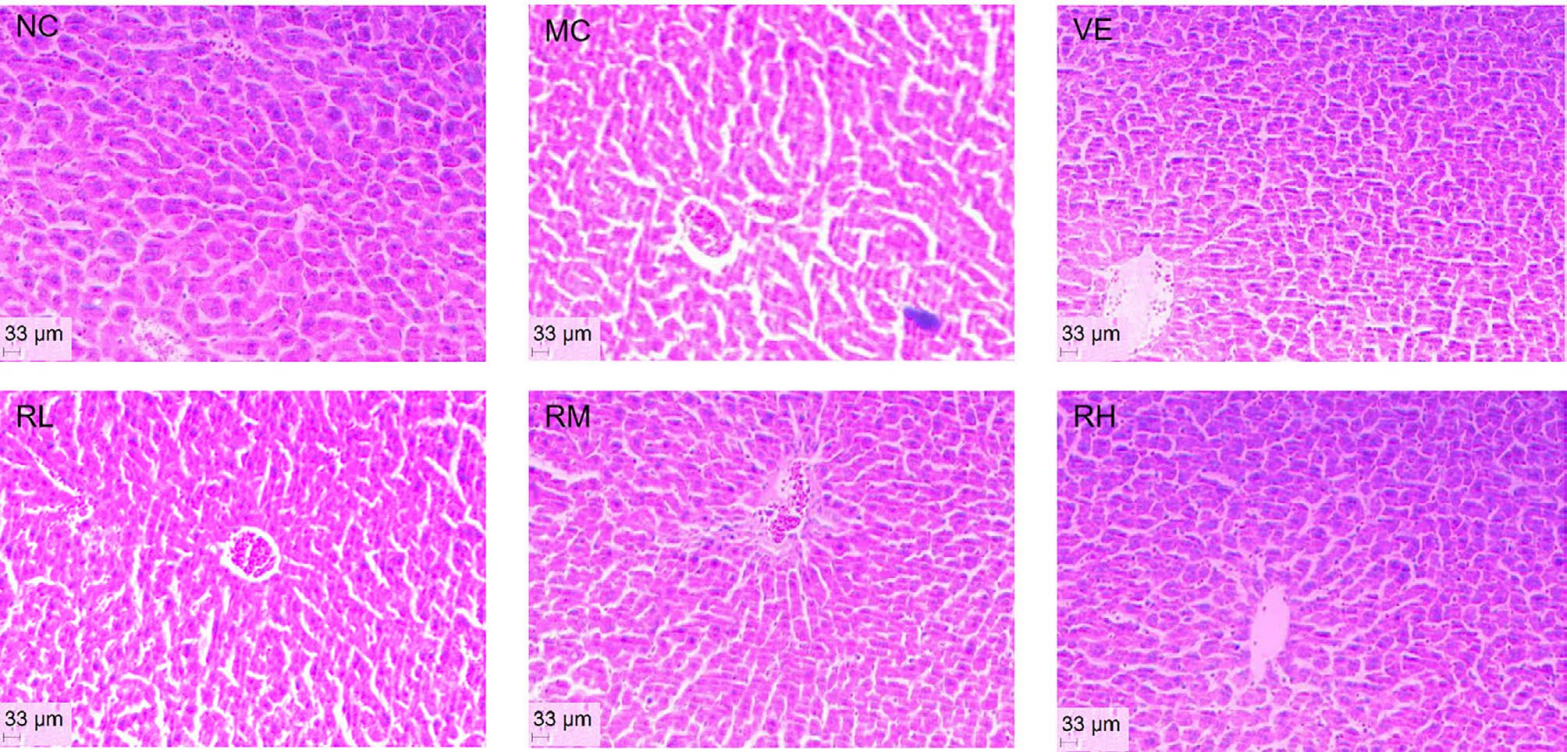
HE staining of mice liver. HE staining, Hematoxylin–eosin staining; MC, model group; NC, blank control group; RL, RM, and RH, low‐, medium‐, and high‐dose groups of rhodoxanthin; VE, Positive control group.

HE staining results of mouse liver tissue are shown in Figure 7. The NC group exhibited intact hepatic architecture characterized by well‐defined lobular structures, radially arranged hepatocyte cords around central veins, and nucleoli with typical spherical morphology. In contrast, the MC group demonstrated severe histopathological alterations, loss of distinct lobular boundaries with sinusoidal dilation, chromatin margination, cytoplasmic vacuolization in hepatocytes, and accumulation of lipofuscin granules. Rhodoxanthin intervention dose dependently attenuated these degenerative changes. Notably, the 80 mg/kg RH group and the VE group displayed near‐normal histological profiles, including an improved hepatic lobule structure to some extent, orderly liver cord arrangement, and reduced lipofuscin deposition. These findings demonstrated that RH effectively counteracted D‐galactose‐induced hepatic degeneration, achieving therapeutic efficacy equivalent to the gold standard VE treatment.

**FIGURE 8:**
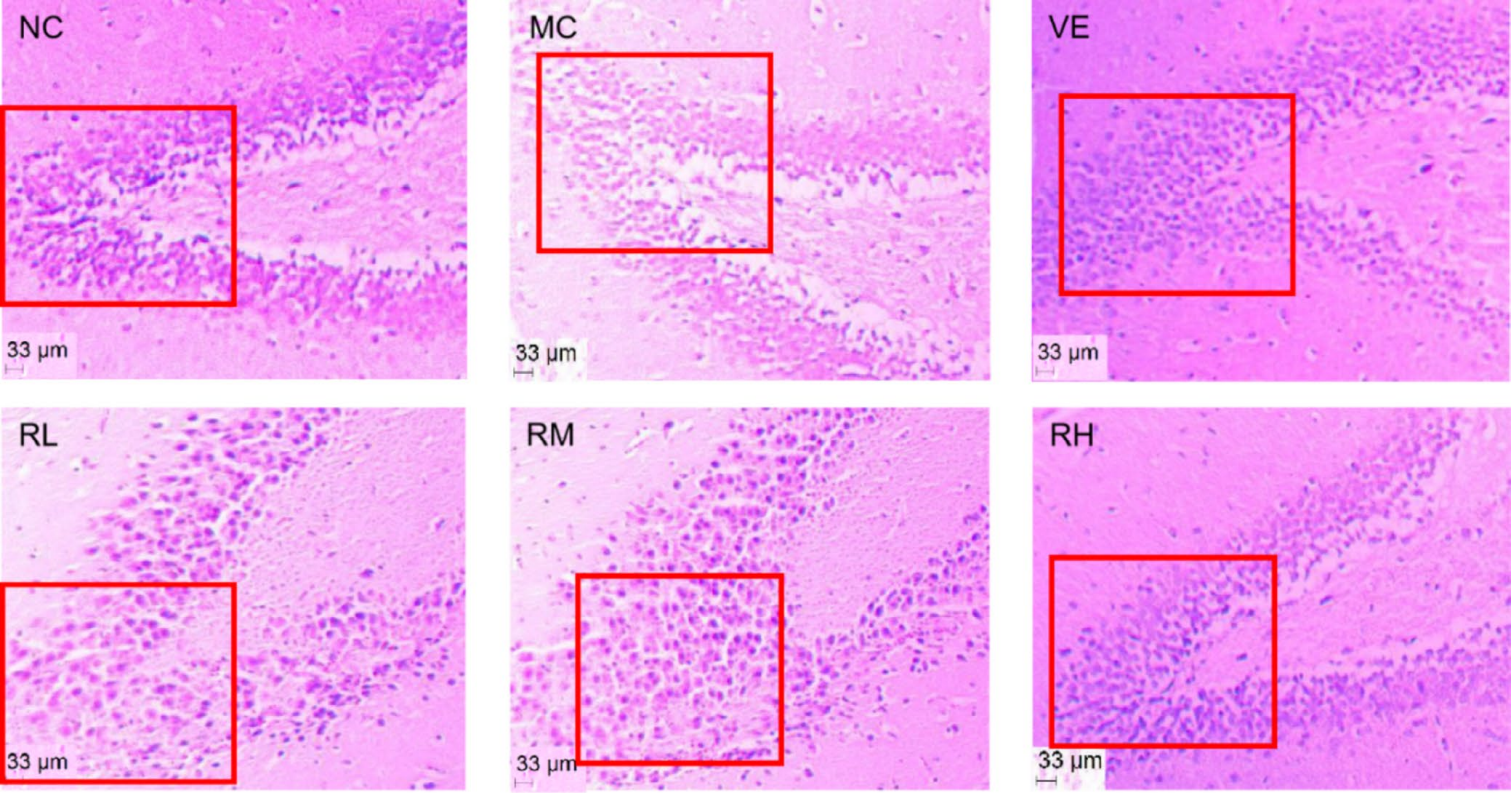
HE staining of mice brain. HE staining, Hematoxylin–eosin staining; MC, model control group; NC, blank control group; RL, RM, and RH, low‐, medium‐, and high‐dose groups of rhodoxanthin; VE, positive control group.

Histopathological evaluation of hippocampal morphology via HE staining was presented in Figure 8. In the NC group, hippocampal neurons were closely arranged, abundant, intact, and without necrosis. In stark contrast, neurons in the hippocampus were sparse, and hippocampal neuron cells were atrophied or lost in the MC group. In the RL and RM groups, hippocampal cells were arranged loosely, and a small extent of necrosis and injury was observed. The hippocampal cells in the RH and VE groups were arranged in a compact and complete shape. These quantitative histomorphometric findings demonstrated rhodoxanthin's dose‐dependent neuroprotective efficacy against D‐galactose‐induced hippocampal degeneration, mechanistically attributable to neurite plasticity enhancement and antiapoptotic regulation.

### 3.5 Effects of Rhodoxanthin on the mRNA Expression of Aging‐Related Genes

**FIGURE 9:**
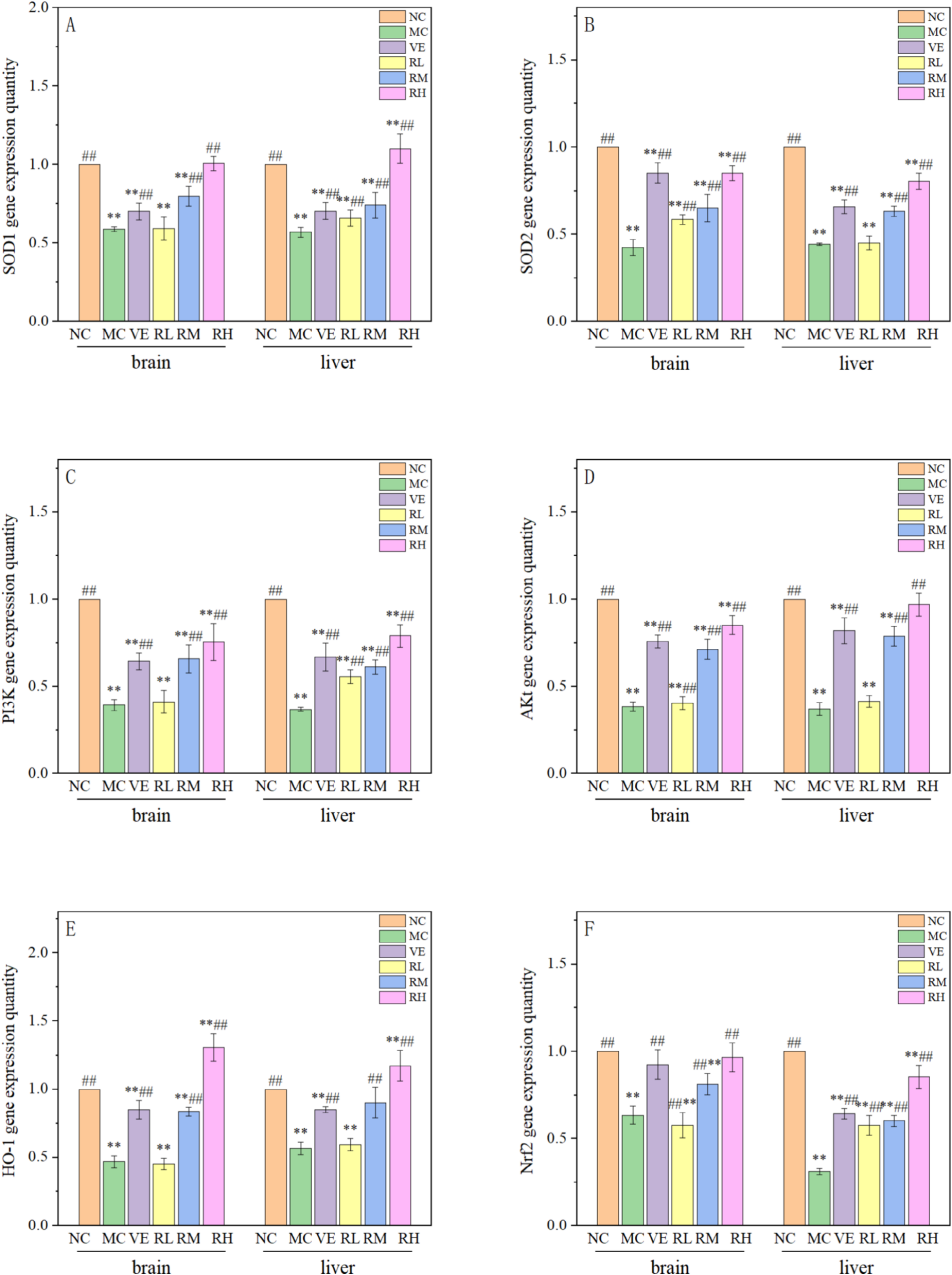
Effects of rhodoxanthin on the expression of target aging‐related mRNA (Nrf2, HO‐1, SOD1, SOD2, PI3K, and Akt) in liver and brain. MC, model control group; NC, blank control group; RL, RM, RH, low, medium and high dose groups of rhodoxanthin; VE, positive control group. Compared with the blank group (NC), *p < 0.05, **p < 0.01; compared with the model group (MC), #*p* < 0.05, ##*p* < 0.01).

In order to further understand the antiaging mechanism of rhodoxanthin, the expression of target aging‐related genes' mRNA (Nrf2, HO‐1, SOD1, SOD2, PI3K, and Akt) was detected in this study (Figure 9).

#### 3.5.1 Effects of Rhodoxanthin on Nrf2 mRNA Expression

Quantitative analysis of hepatic Nrf2 transcriptional regulation is presented in Figure 9, with the NC group normalized to 1.0. D‐galactose‐induced aging mice exhibited significant downregulation of Nrf2 expression in both liver and brain tissues compared to the NC group (*p < 0.01*). The relative expression of Nrf2 gene in liver of the VE group and rhodoxanthin dose groups was increased to different degrees (*p* < 0.01), in a dose‐dependent manner. The RH group had the most significant improvement. This indicated that the relative expression level of the Nrf2 gene in oxidation‐aged mice treated with rhodoxanthin was significantly improved, but the expression level was still lower than that in the normal control group, indicating that the expression of the Nrf2 gene in mice treated with rhodoxanthin was still not fully recovered. At the same time, the improvement effect of the VE group and the RH group in the brain was close to that of the blank control group and had no significant effect, which indicated that RH could significantly improve the Nrf2 gene expression level in the brain of aging mice.

#### 3.5.2 Effects of Rhodoxanthin on HO‐1 mRNA Expression

Effects of rhodoxanthin on the relative expression of the HO‐1 gene are shown in Figure 9. Compared with the NC group, the relative expression of the HO‐1 gene in the liver and brain of mice in the MC group was significantly decreased (*p* < 0.01). The relative expression of the HO‐1 gene in mouse liver increased with the increase of the rhodoxanthin dose, and the VE group was also significantly up‐regulated. The relative expression of HO‐1 genes in the VE, RM, and RH groups was significantly increased compared with the model group (*p* < 0.01). The results showed that the relative expression of the HO‐1 gene in the liver and brain of D‐galactose‐induced oxidative aging mice was improved to a certain extent.

#### 3.5.3 Effects of Rhodoxanthin on SOD1 mRNA Expression

Effects of rhodoxanthin on the relative expression of the SOD1 gene are shown in Figure 9. Compared with the other groups, the SOD1 gene expression in the MC group was significantly decreased (*p* < 0.01). After treatment with different doses of rhodoxanthin, the gene expression level of mice in the liver and brain was increased to different degrees compared with the MC group and showed a dose‐dependent effect. The relative expression level of the SOD1 gene in the RM group in the liver was significantly higher than that in the NC group (*p* < 0.05). The SOD1 gene expression in the VE group was significantly higher than that in the MC group but lower than that in the RH group. These results indicated that rhodoxanthin could significantly reduce the relative expression of the SOD1 gene induced by D‐galactose.

#### 3.5.4 Effects of Rhodoxanthin on SOD2 mRNA Expression

Effects of rhodoxanthin on the relative expression of the SOD2 gene are shown in Figure 9. Compared with the NC group, the relative expression level of the SOD2 gene in the model group was significantly decreased (*p* < 0.01), and the relative expression level of the SOD2 gene in the VE, RL, RM, and RH groups was significantly up‐regulated (*p* < 0.01). Among them, the RH group had the best effect on the relative expression of the SOD2 gene in aging mice. This showed that the relative expression of the SOD2 gene in the brain of aging mice treated with rhodoxanthin was significantly improved; at the same time, the improvement was more pronounced at higher doses of rhodoxanthin.

#### 3.5.5 Effects of Rhodoxanthin on PI3K mRNA Expression

Effects of rhodoxanthin on the relative expression of the PI3K gene are shown in Figure 9. The relative expression level of the PI3K gene in the MC group was significantly decreased compared with the NC group (*p* < 0.01). With the increase in the rhodoxanthin dose, the relative expression of the PI3K gene was higher than that in the MC group (*p* < 0.01). Activation of the PI3K/AKT/Nrf2 pathway can improve behavioral dysfunction and neurological deficits. This study showed that rhodoxanthin treatment could activate the PI3K/AKT signaling pathway, significantly increase the relative expression of the PI3K gene, and improve oxidative stress in aging mice.

#### 3.5.6 Effects of Rhodoxanthin on Akt mRNA Expression

Effects of rhodoxanthin on the relative expression of AKT gene in liver and brain tissue of aging mice are shown in Figure 9. The relative expression level of SOD2 gene in the MC group was significantly decreased (*p* < 0.01). Compared with the MC group, the expression of AKT gene in VE, RM, and RH groups was increased to varying degrees in liver and brain (*p* < 0.01). The enhancement of AKT gene expression by rhodoxanthin was directly related to its concentration.

### 3.6 Effects of Rhodoxanthin on Protein Expression in Brain of D‐Galactose‐Induced Aging Mice

**FIGURE 10:**
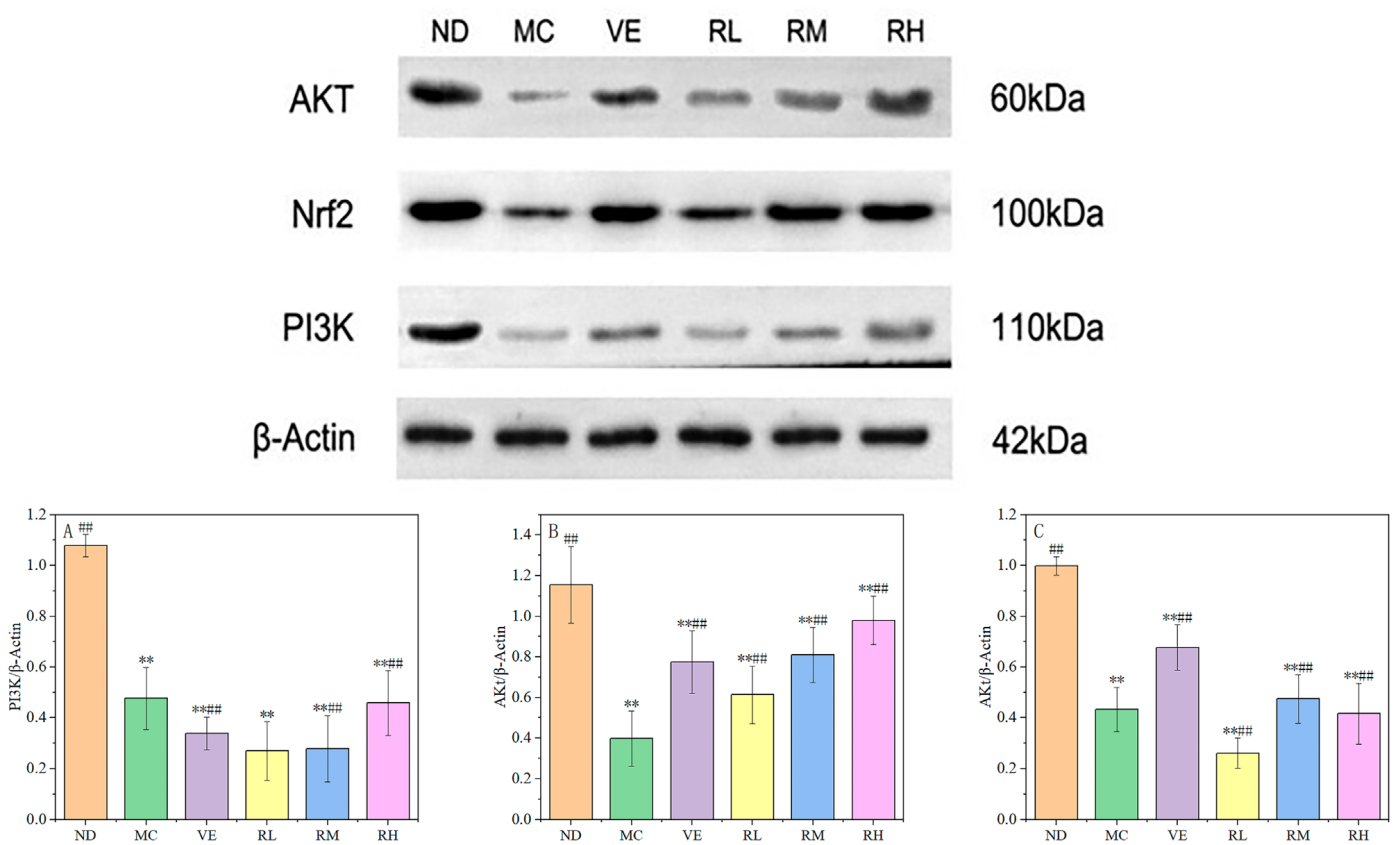
Effects of rhodoxanthin on the protein expression of Nrf2, PI3K, and Akt in the brain of aging mice MC, model control group; NC, blank control group; RL, RM, and RH, low‐, medium‐, and high‐dose groups of rhodoxanthin; VE, positive control group. Compared with the blank group (NC), *p < 0.05, **p < 0.01; compared with the model group (MC), #*p* < 0.05, ##*p* < 0.01).

To further understand the antiaging mechanism of rhodoxanthin, western blotting was used to analyze the expression of target molecular proteins (PI3K, AKT, and Nrf2). As demonstrated in our experimental findings, rhodoxanthin treatment dose‐dependently upregulated Nrf2 protein expression in the brains of oxidative stress‐induced aging mice (*p < 0.01*), suggesting its potential to activate the Nrf2‐mediated antioxidant defense system and mitigate age‐related neuropathology. Further mechanistic analysis (Figure 10) revealed differential regulation of PI3K/AKT pathway components. The RM, RH, and VE groups significantly elevated both PI3K and AKT protein levels compared with the MC group (*p* < 0.01), and the expression level of AKT protein was significantly upregulated in the RL group (*p* < 0.01), without altering PI3K levels (*p > 0.05*). Results indicated that rhodoxanthin could activate the PI3K/AKT signaling pathway, which in turn enhances Nrf2 expression and prevents oxidative stress.

## 4 Discussion

Oxidative stress, resulting from excessive accumulation of ROS beyond physiological levels, represented a key mechanism underlying the aging process. Various exogenous factors including sleep deprivation, alcohol consumption, and ultraviolet radiation have been demonstrated to promote ROS generation (Fitsiou et al. 2021). Excess ROS would oxidize various biological macromolecules, thus disrupting the homeostasis in neurons and eventually leading to the death of related cells (Luo et al. 2020). In this context, natural antioxidants have emerged as promising therapeutic candidates for mitigating oxidative damage and potentially decelerating aging progression.

Previous investigations identified rhodoxanthin, a red carotenoid pigment, as a potent free radical scavenger capable of attenuating H_2_O_2_‐induced cellular damage. Specifically, rhodoxanthin treatment reduced GSH depletion, LDH activity, MDA content, and intracellular ROS levels in oxidative stress models (Fu et al. 2023). Furthermore, it upregulated the expression of antioxidant‐related genes including Nrf2, heme oxygenase‐1 (HO‐1), superoxide dismutase 1 (SOD1), and SOD2 in RAW264.7 cells (Fu et al. 2023). Building upon these findings, the current study demonstrated that chronic rhodoxanthin administration ameliorated D‐galactose‐induced aging in mice through modulation of oxidative damage, lipid metabolism, and the PI3K/Akt/Nrf2 signaling axis, suggesting potential neuroprotective effects.

The D‐galactose‐induced aging model was employed in this investigation due to its well‐established reproducibility, cost‐effectiveness, and high survival rates (Kong et al. 2018). Our experimental design involved 40‐day concurrent D‐galactose injections and rhodoxanthin treatments. Successful model establishment was confirmed by characteristic biochemical alterations: elevated MDA levels, decreased activities of catalase (CAT), superoxide dismutase (SOD), and glutathione peroxidase (GPX), along with reduced expression of Nrf2, HO‐1, SOD1, SOD2, PI3K, and Akt. Remarkably, rhodoxanthin administration, particularly at 80 mg/kg, effectively normalized these perturbations (Figures 6, 7, 8, 9), indicating its capacity to counteract ROS‐mediated cellular damage.

The present study specifically examined rhodoxanthin's modulation of the Nrf2 and PI3K/Akt pathways. Nrf2 serves as a master regulator of cellular redox homeostasis, governing diverse metabolic processes including redox balance, energy metabolism, iron homeostasis, and amino acid metabolism (Liu et al. 2023). Under physiological conditions, Nrf2 remains sequestered in the cytoplasm through binding with Kelch‐like ECH‐associated protein 1 (Keap1). Oxidative stress induced conformational changes in Keap1, facilitating Nrf2 nuclear translocation and subsequent binding to antioxidant response elements (ARE) to activate cytoprotective gene expression (Su et al. 2023). Importantly, our results suggested that rhodoxanthin enhances Nrf2 activity through PI3K/Akt pathway activation, thereby protecting against D‐galactose‐induced oxidative stress.

**FIGURE 11:**
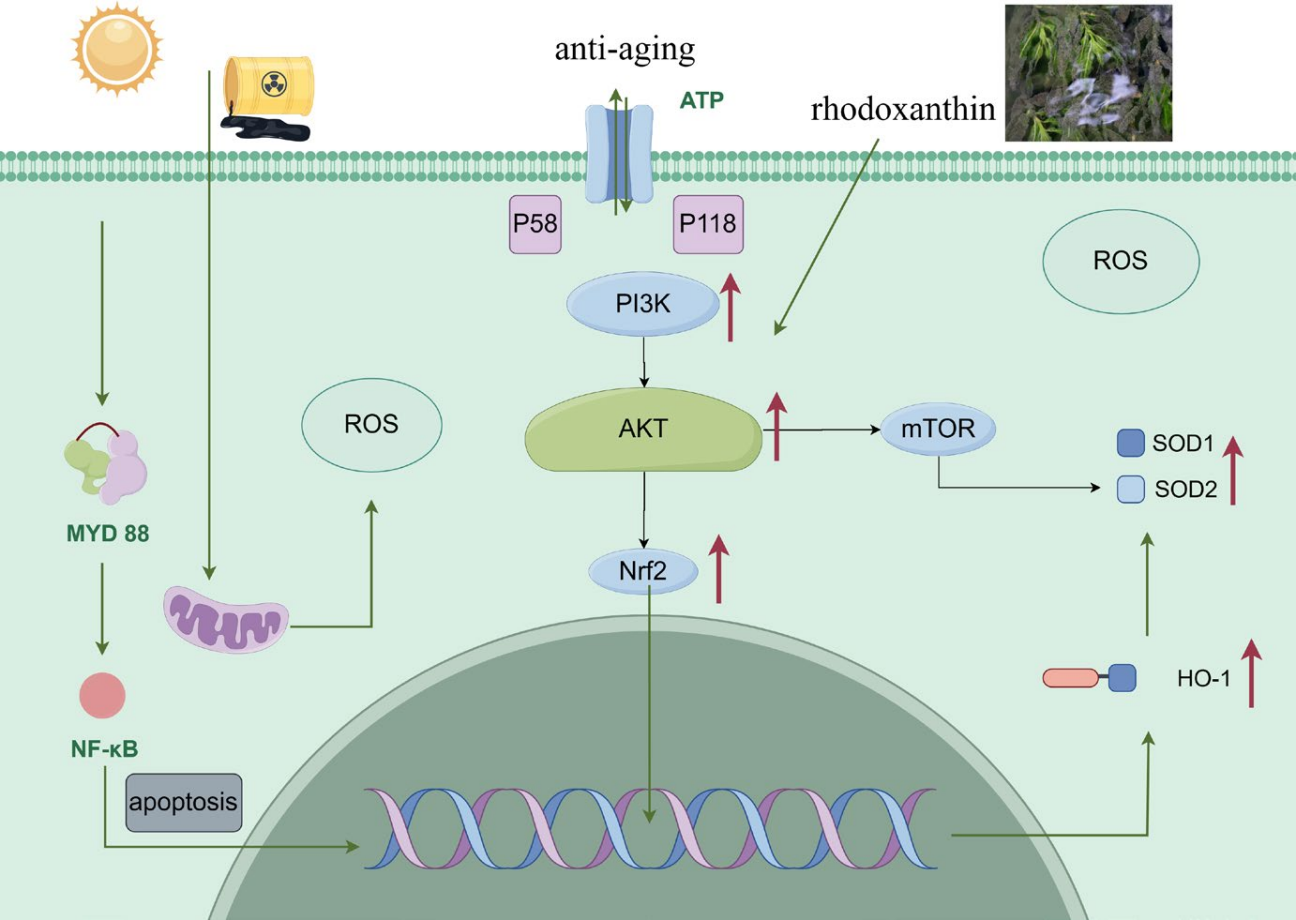
Preliminary study on the mechanism of antiaging.

The mechanistic interplay between these pathways involved PI3K‐mediated generation of phosphatidylinositol (3,4,5)‐trisphosphate (PIP3), which recruited and activated Akt through phosphorylation (Savova et al. 2023). As Nrf2 represented a downstream target of Akt, PI3K/Akt pathway activation promoted Nrf2 nuclear translocation while ROS inhibition conversely suppressed Akt activity (Paraiso et al. 2020). This reciprocal regulation established a feedback loop wherein rhodoxanthin might exert organoprotective effects through PI3K/Akt/Nrf2 pathway activation (Figure 11).

This study has several limitations that warrant consideration. First, while the observed crosstalk between the Nrf2 and PI3K/Akt pathways provides mechanistic insights, deeper molecular validation through functional experiments is required to establish causal relationships beyond correlative gene/protein expression analyses. Second, the exclusive reliance on the D‐galactose‐induced aging model necessitates complementary validation in natural aging paradigms and alternative oxidative stress models to confirm the broad applicability of findings. Finally, the clinical translatability remains constrained by the absence of human‐relevant models and undefined pharmacokinetic‐pharmacodynamic relationships across species, highlighting the need for allometric scaling approaches to optimize human‐equivalent dosing regimens. Collectively, these limitations delineate critical pathways for future investigations to strengthen the therapeutic potential of rhodoxanthin in age‐related disorders.

Our findings demonstrated that rhodoxanthin attenuates aging‐associated oxidative damage through two complementary mechanisms: (1) upregulation of antioxidant genes (Nrf2, HO‐1, SOD1, and SOD2) and (2) activation of the PI3K/Akt signaling cascade. These coordinated actions significantly improved the aging phenotype, highlighting rhodoxanthin's potential as a therapeutic agent for age‐related disorders.

## 5 Conclusion

This study investigated the therapeutic potential of rhodoxanthin against oxidative damage using a D‐galactose‐induced aging mouse model. Our findings demonstrate that rhodoxanthin administration significantly improved organ function indices in the heart, liver, brain, and kidney tissues of aging mice, while concurrently reducing pathological manifestations. The compound effectively maintained antioxidant enzyme activities in hepatic and cerebral tissues and suppressed lipid peroxide accumulation.

Molecular analyses revealed that rhodoxanthin treatment markedly upregulated the expression of key oxidative stress‐related genes, including Nrf2, HO‐1, SOD1, SOD2, PI3K, and Akt, in both liver and brain tissues. Notably, the most pronounced therapeutic effects were observed at a dosage of 80 mg/kg rhodoxanthin.

Mechanistically, we established that rhodoxanthin exerts its neuroprotective effects through activation of the PI3K/Akt signaling pathway, which subsequently enhances Nrf2 expression. This dual modulation of both PI3K/Akt and Nrf2 pathways underlies the compound's efficacy in mitigating age‐related oxidative damage, particularly in cerebral tissues. Dosage and bioavailability in humans may differ significantly from murine models. Further clinical studies are needed.

## Author Contributions

**Yichao Ma:** writing – original draft (equal). **cong fu:** methodology (equal). **Hongchun Dong:** conceptualization (equal). **Xuhui Lei:** formal analysis (equal). **Yuxin Shi:** methodology (equal). **Yunhai He:** methodology (equal), resources (equal). **Shu Liu:** resources (equal), visualization (equal). **Qiukuan Wang:** resources (equal), visualization (equal). **Dandan Ren:** funding acquisition (equal), writing – review and editing (equal).

## Conflicts of Interest

The authors declare no conflicts of interest.

## Data Availability

The datasets generated for this study are available on request to the corresponding author. The animal experiment was approved by the Ethical Committee of Dalian Ocean University (No: DLOU2023008).
